# Raciborski on Puberty

**Published:** 1845-05

**Authors:** 


*Raciborski on Puberty.*—He next notices (p. 77) cases of extraordinarily precocious menstruation of which he has collected many cases from various authors, and finds in the theory he has adopted with reference to the cause of menstruation, a ready explanation of these exceptional occurrences. Menstruation which is the index of aptitude for generation appears last in the train of changes, and when all the organs have acquired their due development. In some countries these changes take place earlier than in others, and there seems to be no reason for regarding menstruation during infancy as any thing else than a highly exaggerated degree of precocious puberty.

In both cases the premature appearance of the menses seems to depend on the precocious development of the ova and of the Graafian follicles, which give, as we know, the impulse to all the other organs of the reproductive apparatus: only in one case this precocity evidently depends on the influence of climate or temperature, while in the other it appears to be the effect of congenital peculiarities. One circumstance in favor of this opinion is the invariable coincidence of development of the mammæ and external genital organs with the hemorrhage, in cases of premature menstruation. These phenomena, indeed, do not occur until the follicles of Graaf are already on the point of attaining that full degree of development which characterizes the period of puberty; they are absent, or exist only in a rudimentary degree, whenever the development of these follicles is impeded by some primitive disposition of the economy; by diseases, or by the congenital absence of the ovaries, or their removal.

In support of this opinion too, he observes that though the Graafian follicles are usually not formed until after birth, he yet found a perfectly developed Graafian vesicle on one occasion in the ovary of a child still-born at the seventh month of pregnancy.

Now every thing is in favor of the supposition that had this child continued to live, it would have presented several follicles at the conclusion of pregnancy, and it is very probable that if the same degree of vital energy had continued, it would soon have arrived at the end of some months of extra-uterine life; at that state which is not usually attained before the age of fourteen years. That which we observed as an exception to the general rule in the above-mentioned fact, may occur in all cases of premature menstruation. It may be said, indeed, that in all cases of this kind, the most important anomaly consists in the premature development and precocious maturity of the ova; the menstrual hemorrhage is only the necessary consequence of this anatomical and physiological peculiarity."

It must not however be inferred that the converse of this holds good, and that in women in whom the occurrence of menstruation does not take place till a very late period, there is a corresponding delay in the maturity of the ova and Graafian follicles.

"So far indeed, from that being the case, these facts seem only to confirm what we shall hereafter prove more directly, namely, that the menstrual hemorrhage is only a secondary phenomenon attendant on menstruation strictly so called, and that the capital phenomenon of this function consists in the periodical maturity and elimination of ova. There are some women in whom nothing else occurs besides this phenomenon and authentic cases are on record of women who have given birth to several children without having ever menstruated. Still stronger reasons are there for admitting the possibility of those cases in which, after the ovaries have for a length of time performed their monthly function unattended by any other phenomenon, menstrual hemorrhage at length occurs. Many other signs of puberty no less valuable than the flow of the menses serve to indicate the existence of that state when the menstrual flux is absent.

Long continued illness, it is well known, often has the effect of retarding the epoch of puberty, even after some of its precursory symptoms have appeared, or of suppressing the menstrual flux, although it may have already occurred once or twice. In such cases, the development of the Graafian follicles is found to have been arrested, as was observed by M. Raciborski in the case of two chlorotic girls who died between the ages of sixteen and seventeen; and in whom the Graafian vesicles, though as numerous as they should be at the period of puberty, were of extremely small size. In women in good health, however, menstruation when once established continues for the most part to recur regularly every month. Having noted the interval between the first two menstrual periods in eighty-seven women, M. Raciborski found that, in fifty eight, this interval did not exceed one month. In two women the second menstruation followed six weeks after the first; in four, after an interval of two months; in five, after three months, in four, after four months; in one, at the end of five months; in another, at the end of eight months; in three, at the end of one year; and in another, two years had elapsed. From these data it appears that, from the first appearance of menstruation, it recurs regularly every month in about two thirds of all healthy females.

